# Use of the novel curved GOKU balloon catheter for acute-angled lesions in palliative surgery for congenital heart disease: comparison with a conventional straight balloon

**DOI:** 10.1007/s00380-021-01786-2

**Published:** 2021-02-07

**Authors:** Takanari Fujii, Hideshi Tomita, Kota Nagaoka, Takeshi Shimizu, Nobuo Oyama, Hiroaki Kise, Suguru Tarui, Yoshinori Miyahara, Kozo Ishino

**Affiliations:** Pediatric Heart Disease and Adult Congenital Heart Disease Center, Showa University Hospital, 1-5-8, Hatanodai, Shinagawa-Ku, Tokyo 142-8666 Japan

**Keywords:** Congenital heart disease, Acute-angled lesions, Balloon angioplasty, Curved GOKU

## Abstract

Obstruction develops commonly at the acute-angled portion of the vessels following palliative surgery, such as systemic–pulmonary shunt (SP shunt), right ventricle–to–pulmonary artery shunt (RV–PA shunt) in the Norwood–Sano procedure for hypoplastic left heart syndrome, and cavopulmonary (Glenn) anastomosis. Although balloon angioplasty is a treatment option, dilation with existing straight balloons is sometimes ineffective and technically complicated because of balloon slippage and target vessel distortion. In this study, we investigated the effectiveness of a curved GOKU balloon catheter for balloon angioplasty in postoperative acute-angled lesions associated with palliative surgery for congenital heart disease. We reviewed patients who underwent balloon angioplasty for angled lesions complicated by SP shunt, RV–PA shunt, or Glenn anastomosis, using the novel curved GOKU or a conventional balloon catheter, such as a Sterling balloon catheter. We evaluated patients’ backgrounds, balloon specifications, target lesion anatomical features and angles, and short-term outcomes. We evaluated 45 procedures in 18 patients. A curved GOKU was used in 20 procedures, and a Sterling balloon in 25 procedures. The angulation of the lesions at maximum balloon inflation was significantly smaller using a curved GOKU vs a Sterling balloon [70–120 (mean ± standard deviation, 97 ± 40) degrees vs 110–180 (149 ± 46) degrees, respectively; *p* < 0.001], while the original angle was similar between the groups. Patients’ short-term outcomes with the curved GOKU were excellent, with a significantly better percent increase in minimum lumen diameter of 0–220% (92% ± 66%) vs 0–46% (18% ± 15%) with the Sterling balloon (*p* < 00.1) and with less frequent balloon slippage. The curved GOKU was more effective in balloon angioplasty for acute-angled lesions compared with a conventional straight balloon, likely because of better conformability to the lesion angle and slip resistance.

## Introduction

**Fig. 1:**
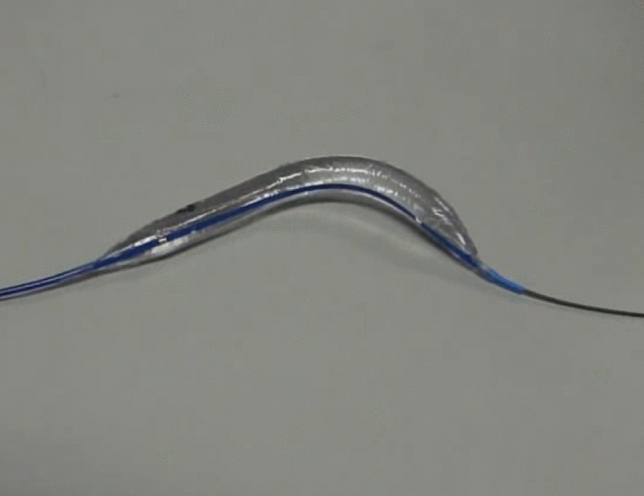
Curved GOKU balloon catheter (at maximum inflation)

Obstruction develops commonly at the acute-angled portion of the vessels following palliative surgery, such as systemic–pulmonary shunt (SP shunt) [1–3], right ventricle–to–pulmonary artery shunt (RV–PA shunt) in the Norwood–Sano procedure for hypoplastic left heart syndrome [4–7], and cavopulmonary (Glenn) anastomosis [8, 9]. Catheter intervention is an effective treatment option for these lesions; however, balloon angioplasty using existing straight balloons is sometimes ineffective and technically complicated [10, 11]. In our experience, the technical failure of balloon angioplasty for such lesions is partially caused by balloon slippage and target vessel distortion. Moreover, acute angulation at the proximal and/or distal anastomosis may cause excessive stress on the vessel wall adjacent to the stenosis during the procedure. The curved GOKU balloon catheter (Tokai Medical Products, Aichi, Japan) is specially designed for acute-angled lesions and bends to approximately 90° when maximally inflated (Fig. 1). This is a noncompliant balloon made of polyamide elastomer with a low profile that can pass through a 4-Fr sheath, and that can accept a 0.018-inch guidewire. Balloon diameters are 4, 5, 6, and 8 mm, and the lengths are 2 cm and 4 cm. The nominal and burst pressures are 13 atm and 18 atm for the 2-cm and 4-cm lengths, respectively. We previously reported the results of an in vitro study evaluating vessel wall stress caused by an inflated curved GOKU in a curved vessel model and our initial clinical experience in five cases [12]. The results suggested that this balloon may be a reasonable alternative in angled lesions, providing better conformability and preventing excessive stress to the vessel wall adjacent to the stenosis. Clinically, this balloon conforms easily in the target lesion at inflation, and slippage and distortion of the balloon are unlikely. However, there is a paucity of clinical data comparing the GOKU with conventional straight balloons. The purpose of this study was to investigate the effectiveness of the curved GOKU for balloon angioplasty in postoperative acute-angled lesions associated with palliative surgical treatment in congenital heart disease.

## Materials and methods

We reviewed patients who underwent balloon angioplasty using the curved GOKU vs the Sterling balloon catheter, which is a conventional straight balloon with similar specifications to the curved GOKU, from January 2011 to May 2019 at Showa University Hospital and Showa University Northern Yokohama Hospital. Because the types of target lesions in balloon angioplasty varied in our patients, we extracted data of patients undergoing SP shunt, RV–PA shunt, or Glenn anastomosis for analysis, to clarify the effects of the differences between the two balloon types. The indications for balloon angioplasty were: (1) progressive desaturation or (2) scheduled unclipping of a previously-clipped conduit for SP shunt and RV–PA shunt; while for Glenn anastomosis, the indications were: (1) unexpected low oxygen saturation, (2) increased superior vena cava pressure, or (3) significant laterality of pulmonary blood flow. In SP shunt and RV–PA shunt, we selected a balloon diameter 1–2-mm larger than the diameter of the expanded polytetrafluoroethylene (ePTFE) graft used for the previous surgery, while in the Glenn anastomosis, we used a balloon measuring either 1–3 times to than the minimal lumen diameter, or not exceed 1.5 times of the reference vessel diameter. The curved GOKU was selected by operator’s preference based on the initial clinical experience [12].

We evaluated patients’ backgrounds, balloon specifications, target lesion anatomical features and angles, and acute outcomes. Regarding the anatomical features of the target lesions, we evaluated the minimum lumen diameter (MLD) of the target lesion and percent diameter stenosis (% DS), defined as [(reference vessel diameter-MLD)/reference vessel diameter] × 100, before and after the procedure, and the % increase in MLD, defined as [difference in MLD before and after procedure]/MLD, before the procedure. We defined the angle of the lesion as the angle formed by the proximal and distal vessels of the stenosis. The original angle of each lesion and the angle change at balloon inflation were measured on the angiograms using the most appropriate angled views; i.e., lateral view for RV–PA shut, and the frontal or left or right anterior oblique view for SP shunt or Glenn anastomosis. We also evaluated balloon slippage or displacement at inflation, which affects the effective dilation of the lesion. We compared these data between the two balloon catheter groups.

Each parameter is expressed as mean ± standard deviation, and we used Wilcoxon’s rank-sum test to compare means between each variable. The chi-square test was used to evaluate the incidence of balloon slippage between the groups, and statistical analyses were performed using JMP® 10 (SAS Institute Inc., Cary, NC, USA). *p *values < 0.05 were considered statistically significant. This study was approved by the Showa University Ethics Committee (Approval number: 3209).

## Results

**Fig. 2:**
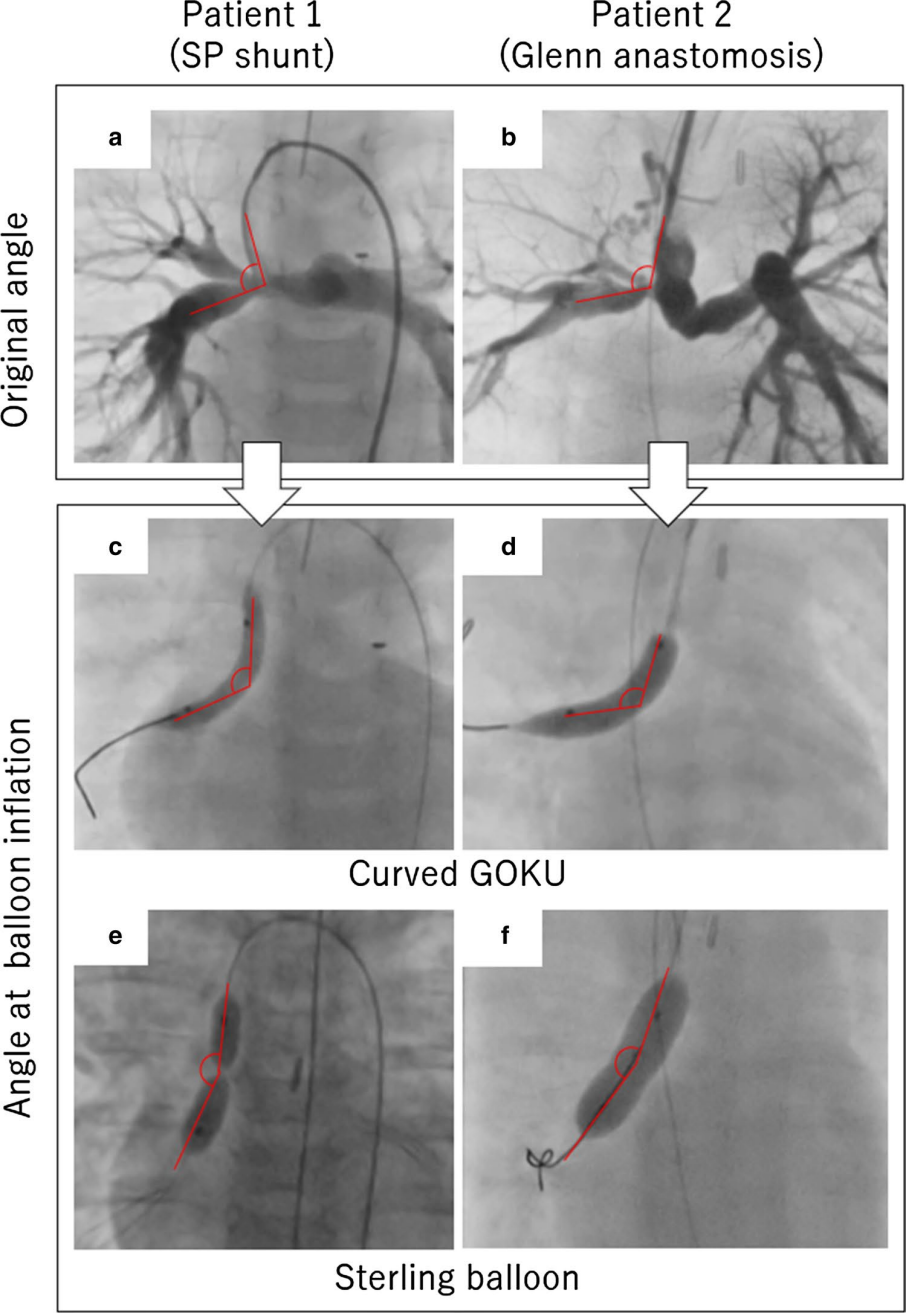
Two representative cases before and at the time of balloon dilation with a curved GOKU vs a Sterling balloon. The lines in the figure show the angle of the lesions. **a** Angiogram in patient 1 showing stenosis in an SP shunt. **b** Angiogram in patient 2 showing stenosis in the right pulmonary artery at the sight of a Glenn anastomosis. **c** Balloon dilation with a curved GOKU in patient 1. **d** Balloon dilation with a curved GOKU in patient 2. **e** Balloon dilation with a Sterling balloon in patient 1. **f** Balloon dilation with a Sterling balloon in patient 2

**Fig. 3:**
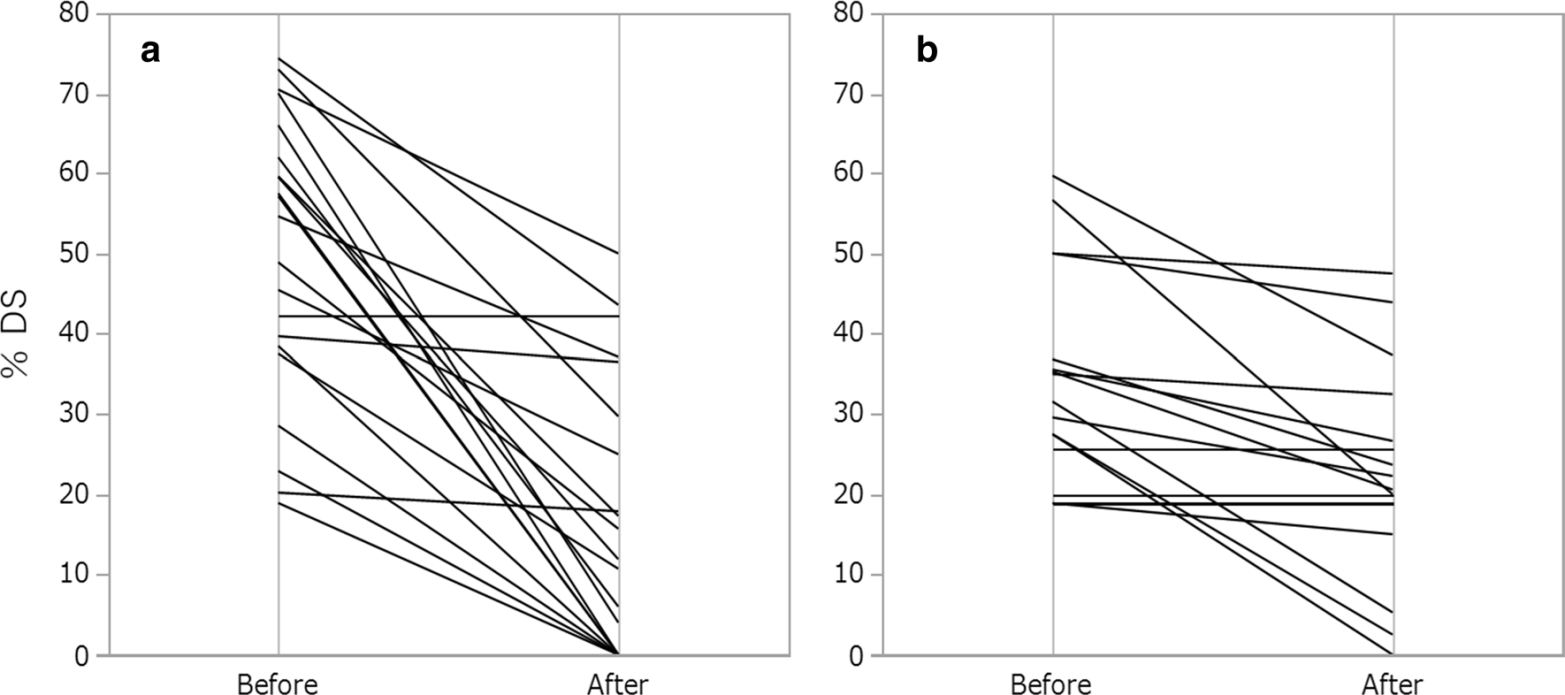
Percent diameter stenosis before and after the procedures in each group. **a** Curved GOKU group. **b** Sterling group

We evaluated 45 procedures in 18 patients. A curved GOKU was used in 20 procedures, and a Sterling balloon was used in 25 procedures. Underlying heart diseases were pulmonary atresia with ventricular septal defect (*n* = 5), hypoplastic left heart syndrome (*n* = 4), pulmonary atresia with the intact ventricular septum (*n* = 2), congenitally corrected transposition of the great arteries (*n* = 2), and tricuspid atresia, complete transposition of the great arteries, single left ventricle, double outlet right ventricle, and coarctation of the aorta with subaortic stenosis (*n* = 1 for each). Target lesions were associated with SP shunt in 10 patients, Glenn anastomosis in 5 patients, and RV–PA shunt in 3 patients. Details of the patients’ backgrounds, balloon specifications, target lesion anatomical features and angles, and short-term outcomes are shown in Table 1. The balloon diameter in the two groups ranged 4–8 mm for the curved GOKU and 4–10 mm for the Sterling balloons. Scheduled unclipping of a previously-clipped ePTFE graft was performed in 3 patients and 1 patient (GOKU and Sterling), respectively. In seven patients who underwent balloon angioplasty using a Sterling balloon, because significant balloon slippage occurred in five patients, and inadequate dilation owing to balloon kinking occurred in two patients, we replaced the Sterling balloons with a curved GOKU in the same session. Patients’ ages and weights at the time of the procedure and the category of the lesions were similar in both groups. The angulation of the lesions at maximum balloon inflation was significantly smaller in the curved GOKU group [70–120 (mean ± standard deviation, 97 ± 40) degrees vs 110–180 (149 ± 46) degrees, in the Sterling group, respectively; *p* < 0.001], while the original angle was similar in both groups. Furthermore, given the significantly small difference of only 12° between the original angulation and that at maximum balloon inflation, the curved GOKU showed better conformability than the Sterling balloon in acute-angled lesions. A representative angiogram and changes in the angles of the lesions at inflation for both balloon types are shown in Fig. 2. Short-term outcomes in patients treated with the curved GOKU were excellent, with a significantly better % increase in MLD of 0–220% (92% ± 66%) vs 0–46% (18% ± 15%) with the Sterling balloon (*p* < 0.01). The incidence of balloon slippage in the curved GOKU group was 1/20 while 8/25 in the Sterling balloons group, which was not significant but tend to be lower in the curved GOKU (*p* = 0.07) (Table 1; Fig. 3). The curved GOKU was well engaged in the lesions without balloon slippage except in one procedure. The slip mechanisms observed in the Sterling group, which were overcome in the curved GOKU group, were obvious balloon kinking inside the distorted angled lesion and milking at an ePTFE graft involved in the stenosis. No procedure-related complications were observed in either group except for balloon slippage.

**Table 1:** Patient characteristics, balloon size, angle of the lesions, and outcomes

Characteristic	Curved GOKU (*n* = 20)	Sterling (*n* = 25)	*p *value
Age (years)	1.6 ± 1.7	0.9 ± 0.5	ns
Weight (kg)	8.3 ± 3.4	7.0 ± 2.3	ns
Category (*n*)			
SP shunt	11	15	
RV–PA shunt	5	7	
Glenn anastomosis	4	3	
Balloon diameter (mm)/No. of lesions	4/1	4/5	
	5/6	5/7	
	6/10	6/5	
	8/3	7/1	
		8/4	
		9/2	
		10/2	
Angle of the lesion			
Original	85 ± 19°	85 ± 17°	ns
At balloon inflation	97 ± 40°	149 ± 46°	< 0.001
MLD (mm)			
Before	2.5 ± 1.2	2.9 ± 1.3	ns
After	4.2 ± 1.1	3.8 ± 1.2	ns
% DS (%)			
Before	49 ± 20	29 ± 22	
After	9.8 ± 26	19 ± 20	
% Increase in MLD (%)	92 ± 66	18 ± 15	< 0.01
Balloon slippage (*n*)	1/20	8/25	0.07

*MLD* minimum lumen diameter, *RV–PA shunt* right ventricle–to–pulmonary artery shunt, *SP shunt* systemic–pulmonary shunt, *%DS* percent diameter stenosis

## Discussion

We previously assessed the wall stress distribution in a curved vessel model, and found that the curved GOKU naturally curved to the lesser curvature and generated uniform stress on both the greater and lesser curvatures, whereas the straight balloons caused disproportionate stress on each side of the wall [12]. In the initial report evaluating the curved GOKU, all procedures were completed because the curved balloon naturally conformed well to the angled lesion without slipping, while the authors speculated that the curved GOKU may reduce the risk of vessel damage associated with disproportionate stress caused by straight balloons [12]. In the current study, our findings suggested that the curved GOKU is advantageous not only in reducing complications but also in contributing to better short-term outcomes. We also previously examined the possibility of stenting with a curved balloon for angled lesions in vitro and in an animal model, and reported that post-dilation to conform the stent to the angled vessel would be a reasonable option [13]. In the current study, the anatomical properties of the lesions before the procedure, MLD, % DS, and the original angle of the lesions were similar in the two groups. Therefore, the difference in acute outcomes was likely because of differences in the performance of the balloons.

To our knowledge, ours is the first study emphasizing the superiority of the curved GOKU over conventional straight balloons in angioplasty for postoperative acute-angled lesions. The curved balloon provided short-term acute outcomes with a significantly better % increase in MLD and less angle-alternation of the lesion during balloon inflation. According to these results, we speculated that excellent acute outcomes with the curved GOKU may result from better balloon conformability to the angle of the lesions and better engagement with slip resistance.

Determining which degree of acuteness of the original angle of the lesion is reasonable for using a curved GOKU, with its obvious advantages, is a concern. In our patients, the original angles of the lesions ranged from 70 to 110 (mean, 85) degrees in the groups. In bench testing, the curved angle of the curved GOKU is 90°, 88°, and 80° at 10, 13, and 18 atmospheres, respectively [12, 13]. Given this property of the balloon and the patients’ lesion angles, we consider the curved GOKU may be particularly useful for lesions more acute than approximately 90°. Regarding balloon slippage, the contributing factors may include lesion substrate (native tissue or ePTFE graft), balloon size relative to MLD, and balloon shape (curved or straight). In eight cases with significant balloon slippage with the Sterling balloon, seven cases were associated with an ePTFE graft, five with SP shunt, and two with RV–PA shunt. Therefore, in angled lesions associated with ePTFE, the curved GOKU has an advantage. From these data, we suggest that the recommend indication for using a curved GOKU rather than a conventional straight balloon are: (1) lesion more acute than 90°, or (2) acute lesions associated with an ePTFE graft.

Regarding the disadvantage of this balloon, there is some limitation in rewrapping, which may cause difficulty to re-cross the stenosis. Acceptable but a little bit lower trackability compared to the Sterling balloon may be another limitation. Although no objective data is available associated with industrial secrets, we suppose these limitations are caused by its special characteristic of balloon wrapping to bend when inflated.

There are limitations in this study. First, measurement errors in the angles based on two-dimensional angiograms of the three-dimensional lesions are possible. Second, we did not compare differences between the two balloons for the same lesion, and with identical balloon specifications (diameter, length, and pressure). Finally, our patient numbers were low.

## Conclusions

Using a curved GOKU for angled lesions may allow for better conformability and prevent slippage at balloon inflation. The curved GOKU is more effective for balloon angioplasty in acute-angled lesions compared with a conventional straight balloon.
